# Support of Medical Schools

**Published:** 1913-04

**Authors:** 


### OUR CONTEMPORARIES.

### Support of Medical Schools.

The question of adequate support for medicical education for the next decade is a most far reaching and important one. Increased funds of varying amounts have been widely procured for laboratories, hospital extensions, and for the promotion and full teaching and research. The Education Department of New York has taken advantage of this improvement to announce that in the near future it will cease to recognize schools employing less than six whole-time instructors. The department, furthermore, very wisely refuses to recognize nominal salaries as complying with its conditions. Naturally, in this general effort to procure more money in order to escape furtther criticism, waste inevitably occurs; many schools which would do better to emulate the example of those who have closed their doors struggle to imitate those that are destined to survive. Gifts and appropriations of $5,000 and similar sums for improvements only enable weak establishments to maintain themselves a little longer. In no field of education today can wise giving do more than in medicine, but in no field does the intending giver need wiser discrimination.—Editorial in North Tonawanda News, March 11, 1913.

While we agree in the main with the above statement, it should be remembered that a gift of $5,000 to an existing institution, of any kind, whose status and accomplishments can be investigated, will probably have as great efficiency as $10,000—or perhaps $50,000—comprising part of a large sum for the establishment of a new institution. It is no discredit to a medical school that it needs assistance. Under present conditions there is not a medical school in the country that exists or can exist on the basis of a business organization, that is, that can pay running expenses and interest on investment (unwatered) from the fees of students. The true philanthropist would do well to study the ratio of per capita cost of instruction to fees; to ask whether the institutions under consideration have done and are doing conscientious work; and, very frankly, to ask how far efficiency and how far glory enters into the motives of donor and executives.

---

*The Canadian Journal of Med. and Surg.* for March is copiously illustrated. The editor strongly favors the principle of British Liberty, objecting to certain arbitrary commands of school inspectors, as that a child be excluded from school because of the objection of the child, his family and his dentist, to having the entire set of deciduous teeth extracted; or until the tonsile have been extirpated, especially in a case in which they had already been removed. Personal liberty, by the way, used to be quite a hobby in the U. S.

---

*The Medical Review* of St. Louis has incorporated the *Medical Era*. The *Review* is one of our brightest exchanges, and we take pleasure in crediting its able management to a woman's intellect.

